# Antimicrobial Resistance: Addressing a Global Threat to Humanity

**DOI:** 10.1371/journal.pmed.1004264

**Published:** 2023-07-03

**Authors:** Timothy R. Walsh, Ana C. Gales, Ramanan Laxminarayan, Philippa C. Dodd

**Affiliations:** 1 Ineos Oxford Institute for Antimicrobial Resistance, Department of Zoology, Oxford, United Kingdom; 2 Division of Infectious Diseases, Escola Paulista de Medicina, Universidade Federal de São Paulo (EPM-UNIFESP), São Paulo, Brazil; 3 Princeton University, Princeton, New Jersey, United States of America; One Health Trust, Bengaluru, India; 4 Public Library of Science, San Francisco, United States of America and Cambridge, United Kingdom

Antimicrobial resistance (AMR) has been prioritized by the World Health Organization (WHO) as one of the top 10 global public health threats facing humanity [1]. The High-Level Meeting of the UN General Assembly on Antimicrobial Resistance in 2016 officially declared the importance of AMR and solicited countries to commit to their individual AMR National Action Plans [2]. Despite these efforts, drug-resistant infections were estimated to contribute to a devastating 4.95 million deaths globally in 2019, with the bulk of the clinical burden borne by low- and middle-income countries (LMICs), particularly in sub-Saharan Africa [3]. This far exceeds the annual global deaths attributable to tuberculosis (1.5 million), malaria (643,000) and HIV/AIDS (864,000). Without intervention it is estimated that global deaths attributable to AMR could reach 10 million annually by 2050 [4]. Here, we discuss the Special Issue commissioned by *PLoS Medicine* dedicated to AMR. These research studies affirm the complexity and multi-faceted dynamics of AMR and the enormous challenge faced in understanding the problem and in designing tractable, equitable and cost-effective interventions to control its spread.

The drivers of AMR are multifactorial but there is no debate that antibiotic overuse has been paramount. Between 2000 and 2015 antibiotic use increased by 65% globally, primarily driven by a substantial increase across LMICs [5]. Bacteria are complex organisms that receive and transfer DNA at an alarming frequency with seemingly little or no cost to themselves; and, contrary to previous dogma, the presence of an antibiotic has little impact on the transfer of mobile bacterial DNA but can select for newly acquired resistant mechanisms [6]. AMR is a One-Health problem, and can spread via humans, animals (domestic and wild), and the environment (water and air). Inadequate access to water, sanitation, and hygiene (WASH) as well as inadequate access to healthcare services (e.g., cost-effective diagnostics) and affordable, appropriate antibiotics have served to accelerate the spread of AMR in LMICs. AMR is a critical public health crisis and a One-Health priority requiring engagement across human, agricultural, and environmental sectors.

The Special Issue attracted several studies focused on neonatal and pediatric infections. Neonates and infants are particularly vulnerable to antibiotic resistant infections. In a global prospective study of neonatal sepsis (NeoOBS), which included 11 countries spanning 4 continents, Neal Russell and colleagues identified *Klebsiella pneumoniae* as the most common causative pathogen for neonatal sepsis, associated with mortality rates of 10–12% and higher when blood cultures were positive [7]. In the cohort studied, over 200 different antibiotic combinations were prescribed for 3,204 cases of neonatal sepsis, with significant deviation from WHO-recommended regimens and high usage rates of last resort and reserve antibiotics such as colistin [7]. These data reiterate the need for urgent trials of novel empiric antibiotic regimens to minimize AMR [7]. Carbapenem-resistant *K*. *pneumoniae* (CRKP) has spread to all parts of the world and is a leading cause of drug-resistant infections in neonates and children in LMICs [8]. In a systematic review of over 120 studies across 30 countries including 21 LMICs, Ya Hu and co-workers reported over 140 different sequence types causing neonatal CRKP infections, and among 3 common lineages (ST11, ST15 and ST17), the ST17 lineage was detected in 8 countries across 4 continents, warranting early detection for treatment and prevention [9]. Among the 1,592 neonatal CRKP strains available for analyzing the presence of carbapenemase-encoding genes, NDM (New Delhi metallo-β-lactamase) was the most common carbapenemase identified (64.3%) [9]. The pooled mortality due to neonatal CRKP infections was 22.9% (95% CI 13.0%, 32.9%) [9].

Of course, inappropriate prescribing of antibiotics is not restricted to hospital settings, nor to LMICs. Using data from a prospective, community-based mother-and-child cohort study (the BIRDY cohort) conducted across urban and rural sites in Cambodia, Madagascar, and Senegal between 2012 and 2018, Antoine Ardillon et al. report extensive, inappropriate antibiotic prescribing among pediatric outpatients [10]. The authors identified that 76.5% of consultations resulting in antibiotic prescription were determined not to require antibiotics [10]. Rhinopharyngitis, gastroenteritis and uncomplicated bronchiolitis accounted for the greatest share of inappropriate prescribing, and children older than 3 months living in rural areas were more likely to receive inappropriate prescriptions [10]. These data highlight the importance of implementing local programs to optimize antibiotic prescribing at the community level in LMICs [10]. In a review of the carriage of AMR/AMR genes in children under the age of 2 years, Charlie Luchen and colleagues report that antibiotics significantly impact diversity and composition of the infant gut microbiome in LMICs, while concomitantly selecting for resistance genes which can persist for prolonged periods of time [11]. In LMIC settings, inadequate access to WASH facilitates transmission of AMR gut bacteria between humans, the environment and animals creating a cyclical problem of resistant bacterial transmission. The authors also highlight the considerable heterogeneity in study and sequencing methodology used in current research, limiting our insights into the true impact of antibiotics on the infant gut microbiome [11].

In a systematic review of 109 studies reporting bloodstream infections in hospitalized patients from LMICs, several WHO critical and high priority pathogens were associated with increased mortality, length of hospital stay and admission to intensive care units (ICU), and cost [12]. Wide heterogeneity between WHO regions, income groups, and pathogen-drug combinations was observed [12]. Despite AMR being associated with a substantial disease and economic burden in LMICs, Kasim Allel and colleagues conclude that a paucity of data on bloodstream infections from LMICs hinders implementation of country-specific policies [12]. Countries are required to monitor and record antibiotic use and, using analyses of point prevalence surveys from 99 countries across the globe, the annual number of hospital-associated resistant infections (HARIs) caused by high priority pathogens has been estimated at a staggering 136 million, with the highest burden in China, Pakistan, and India [13]. These estimates highlight the global threat of HARIs and may help define strategies to tackle resistance in hospital settings [13].

In high-income settings, prolonged antibiotic overuse and misuse has driven the development of ‘super-bugs’ that are recognizable to many, such as methicillin-resistant *Staphylococcus aureus* (MRSA). Shortening treatment duration is a common strategy applied by antibiotic stewardship programs in the expectation of reducing the prevalence of antimicrobial resistant bacteria over time. To gain a better understanding of the relationship between treatment duration and prevalence of colonization with antimicrobial resistant bacteria in hospitalized patients, Yin Mo and co-workers applied a model to assess how changing duration of antibiotic treatment would affect the risk of resistance colonization at both individual and population levels [14]. The authors concluded that shortening antibiotic treatment duration may increase or decrease colonization by resistant bacteria, dependent upon individual and combined bacterial and antibiotic characteristics [14]. Healthcare facilities are transmission hot spots for AMR pathogens, fueled by inadequate adherence to appropriate infection control measures. At the beginning of the COVID-19 pandemic, the increased demand for healthcare access and inpatient space alongside disorganized service delivery resulted in unavoidable non-adherence to standard infection control measures. However, the impact on colonization with antibiotic resistant bacteria has not been well investigated or reported. Using a novel mathematical modeling approach, David Smith and colleagues reported that surges in COVID-19 cases fostered conditions favorable for bacterial transmission, on average resulting in a 14% increase in colonization and a 10% increase in rates of AMR [15]. Conversely, the implementation of COVID-19 control measures provided the unintended benefit of limiting bacterial spread, leading to a 28% reduction in patient acquisition of drug-resistant bacteria [15], suggesting that stricter infection control measures are highly likely to be beneficial.

Whilst AMR cannot be neutralized by a single vaccination program, immunization has been well documented to be effective at preventing the spread of infectious diseases, and vaccines targeting resistant microbial species, especially *K*. *pneumoniae*, have been called for by WHO [16]. A modeling study by Chirag Kumar and colleagues examined the impact of a hypothetical *K*. *pneumoniae* maternal vaccine on neonatal sepsis infections and mortality, and estimated that maternal vaccination could avert 80,258 neonatal deaths and 399,015 cases of neonatal sepsis annually worldwide, accounting for more than 3.40% of all neonatal deaths [17]. The largest relative benefits were predicted in Africa (Sierra Leone, Mali, Niger) and South-East Asia (Bangladesh) where vaccination could avert over 6% of all neonatal deaths [17]. Of critical importance is the need for the standardized reporting of AMR infections and antibiotic use, as well as improved diagnostics such that prevalence and resistance patterns at national, regional, and local levels can be ascertained to better inform antimicrobial stewardship programs and appropriate guidelines for antimicrobial use.

In September 2024, a high-level meeting of the United Nations will be held to discuss AMR and to propose an enhanced framework to that outlined in 2016. Recognition of the magnitude of this problem is urgently required by world leaders and policy makers to ensure adequate global investment in the development of innovative affordable vaccines, antimicrobial agents, diagnostics, and reporting systems. The COVID-19 pandemic has taught us, as a global community, that the planet is small and we are all inter-connected economically, culturally, and socially with the ultimate consequence of what happens in one country soon appearing in another. It is estimated that the attributable deaths due to COVID-19 since December 2019 numbered 3.2 million and its cost to the global economy is approximately $17 trillion. On the current trajectory, by 2050, not only will AMR perhaps be responsible for 10 million deaths annually it will also cost the global economy over $100 trillion—figures that dwarf the impact of COVID-19 and emphasize the urgent threat of AMR.

## References

[1] World Health Organization. 10 global health issues to track in 2021. 2020. Available from: https://www.who.int/news-room/spotlight/10-global-health-issues-to-track-in-2021 [Accessed June 11, 2023].

[2] General Assembly of the United Nations. High-Level Meeting on Antimicrobial Resistance. 21 September 2016. Available from https://www.un.org/pga/71/2016/09/21/press-release-hl-meeting-on-antimicrobial-resistance/ [Accessed June 22, 2023].

[3] Antimicrobial Resistance Collaborators. Global burden of bacterial antimicrobial resistance in 2019: a systematic analysis. Lancet. 2022 Feb 12;399(10325):629–655. doi: 10.1016/S0140-6736(21)02724-0 Epub 2022 Jan 19. Erratum in: Lancet. 2022 Oct 1;400(10358):1102. . 35065702 PMC8841637

[4] O’Neill J. Tackling drug-resistant infections globally: Final report and recommendations. The review on antimicrobial resistance. 2016. Available from http://amrreview.org/sites/default/files/160525_Final%20paper_with%20cover.pdf [Accessed June 11, 2023].

[5] Klein EY, Van Boeckel TP, Martinez EM, Pant S, Gandra S, Levin SA, et al. Global increase and geographic convergence in antibiotic consumption between 2000 and 2015. Proc Natl Acad Sci U S A. 2018 Apr 10;115(15):E3463–E3470. doi: 10.1073/pnas.1717295115 Epub 2018 Mar 26. . 29581252 PMC5899442

[6] Orlek A, Anjum MF, Mather AE, Stoesser N, Walker AS. Factors associated with plasmid antibiotic resistance gene carriage revealed using large-scale multivariable analysis. Sci Rep. 2023 Feb 13;13(1):2500. doi: 10.1038/s41598-023-29530-y . 36781908 PMC9925765

[7] Russell NJ, Stöhr W, Plakkal N, Cook A, Berkley JA, Adhisivam B, et al. Patterns of antibiotic use, pathogens, and prediction of mortality in hospitalized neonates and young infants with sepsis: A global neonatal sepsis observational cohort study (NeoOBS). PLoS Med. 2023 Jun 8;20(6):e1004179. doi: 10.1371/journal.pmed.1004179 . 37289666 PMC10249878

[8] Laxminarayan R, Bhutta ZA. Antimicrobial resistance—a threat to neonate survival. Lancet Glob Health. 2016 Oct;4(10):e676–7. doi: 10.1016/S2214-109X(16)30221-2 . 27633421

[9] Hu Y, Yang Y, Feng Y, Fang Q, Wang C, Zhao F, et al. Prevalence and clonal diversity of carbapenem-resistant Klebsiella pneumoniae causing neonatal infections: A systematic review of 128 articles across 30 countries. PLoS Med. 2023 Jun 20;20(6):e1004233. doi: 10.1371/journal.pmed.1004233 . 37339120 PMC10281588

[10] Ardillon A, Ramblière L, Kermorvant-Duchemin E, Sok T, Zo AZ, Diouf JB, et al. BIRDY study group. Inappropriate antibiotic prescribing and its determinants among outpatient children in 3 low- and middle-income countries: A multicentric community-based cohort study. PLoS Med. 2023 Jun 6;20(6):e1004211. doi: 10.1371/journal.pmed.1004211 . 37279198 PMC10243627

[11] Luchen CC, Chibuye M, Spijker R, Simuyandi M, Chisenga C, Bosomprah S, et al. Impact of antibiotics on gut microbiome composition and resistome in the first years of life in low- to middle-income countries: A systematic review. PLoS Med. 2023 Jun 27 doi: 10.1371/journal.pmed.1004235 37368871 PMC10298773

[12] Allel K, Stone J, Undurraga EA, Day L, Moore CE, Lin L, et al. The impact of inpatient bloodstream infections caused by antibiotic-resistant bacteria in low- and middle-income countries: A systematic review and meta-analysis. PLoS Med. 2023 Jun 22;20(6):e1004199. doi: 10.1371/journal.pmed.1004199 . 37347726 PMC10287017

[13] Balasubramanian R, Van Boeckel TP, Carmeli Y, Cosgrove S, Laxminarayan R. Global incidence in hospital-associated infections resistant to antibiotics: An analysis of point prevalence surveys from 99 countries. PLoS Med. 2023 Jun 13;20(6):e1004178. doi: 10.1371/journal.pmed.1004178 . 37310933 PMC10263350

[14] Mo Y, Oonsivilai M, Lim C, Niehus R, Cooper BS. Implications of reducing antibiotic treatment duration for antimicrobial resistance in hospital settings: A modelling study and meta-analysis. PLoS Med. 2023 Jun 15;20(6):e1004013. doi: 10.1371/journal.pmed.1004013 . 37319169 PMC10270346

[15] Smith DRM, Shirreff G, Temime L, Opatowski L. Collateral impacts of pandemic COVID-19 drive the nosocomial spread of antibiotic resistance: A modelling study. PLoS Med. 2023 Jun 5;20(6):e1004240. doi: 10.1371/journal.pmed.1004240 . 37276186 PMC10241372

[16] World Health Organization. Antimicrobial resistance. 2021. Available from https://www.who.int/news-room/fact-sheets/detail/antimicrobial-resistance [Accessed June 16, 2023].

[17] Kumar CK, Sands K, Walsh TR, O’Brien S, Sharland M, Lewnard JA, et al. Global, regional, and national estimates of the impact of a maternal Klebsiella pneumoniae vaccine: A Bayesian modeling analysis. PLoS Med. 2023 May 22;20(5):e1004239. doi: 10.1371/journal.pmed.1004239 . 37216371 PMC10270628

